# Spontaneous Pneumomediastinum and Subcutaneous Emphysema following Cocaine Inhalation and Ecstasy Ingestion

**DOI:** 10.1155/2019/6972731

**Published:** 2019-02-24

**Authors:** Samantha Jaensch, Sang Hwang, Tony Shih-Wei Kuo

**Affiliations:** ^1^Department of Otolaryngology, Gosford Hospital, Gosford, NSW, Australia; ^2^Macquarie University Hospital, Sydney, NSW, Australia

## Abstract

Spontaneous pneumomediastinum (SPM) and subcutaneous emphysema are rare complications of illicit drug abuse. Thorough history, examination, and investigations are required to rule out fatal complications such as oesophageal perforation. We present a case of a 21-year-old male presenting with pleuritic chest pain one day after cocaine inhalation and ingesting ecstasy. Conservative supportive management is appropriate when this occurs spontaneously without radiological evidence of visceral perforation.

## 1. Introduction

Spontaneous pneumomediastinum (SPM) with subcutaneous emphysema (SE) is a rare complication of recreational drug use. It usually follows a benign self-limiting course with supportive management. However, it is crucial to consider serious associated conditions (e.g., pneumothorax or oesophageal perforation) as well as secondary pathological causes.

## 2. Case Presentation

A 21-year-old male with a background of mild childhood asthma presented to ED with sudden onset of chest and neck pain. He had self-administered salbutamol believing he was having an asthma attack, to no effect. The patient denied any trauma or infective symptoms and had no cardiovascular risk factors or past medical conditions. He had been to the gym two days prior where he partook in his usual weightlifting routine and had not engaged in any unusually strenuous activities. However, he did admit to inhaling a small amount of cocaine and ingesting two ecstasy pills (3,4-methylenedioxymethamphetamine, MDMA) approximately 20 hours prior to the onset of symptoms while out dancing with friends.

**Figure 1:**
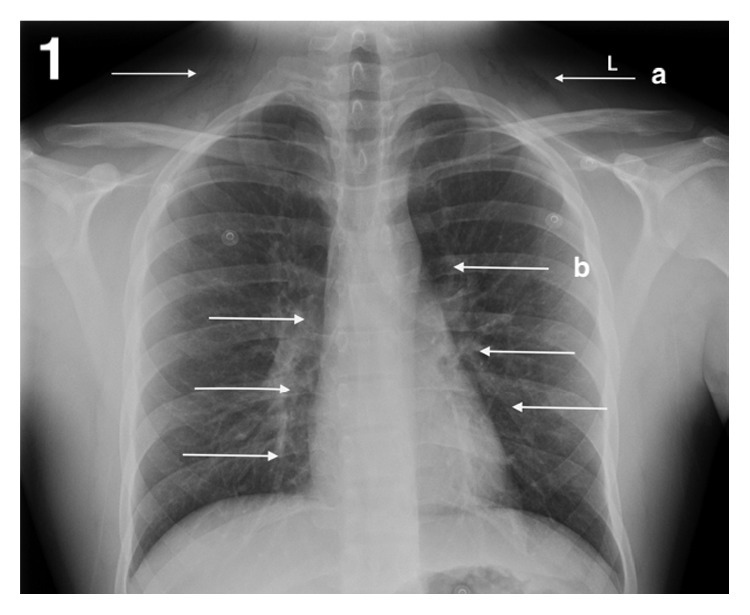
X-ray showing supraclavicular subcutaneous emphysema and mediastinal shadow, consistent with pneumomediastinum.

**Figure 2:**
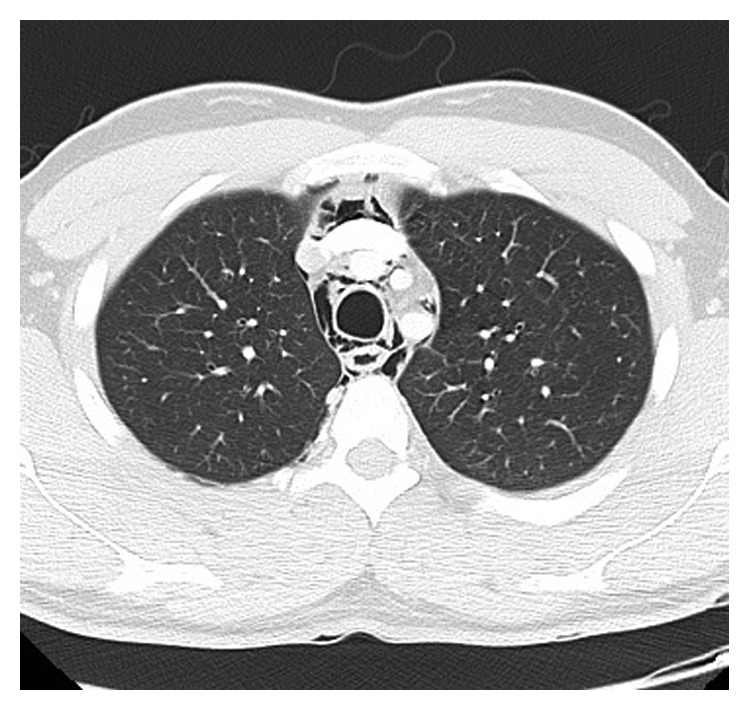
Axial CT showing free air surrounding mediastinal structures.

**Figure 3:**
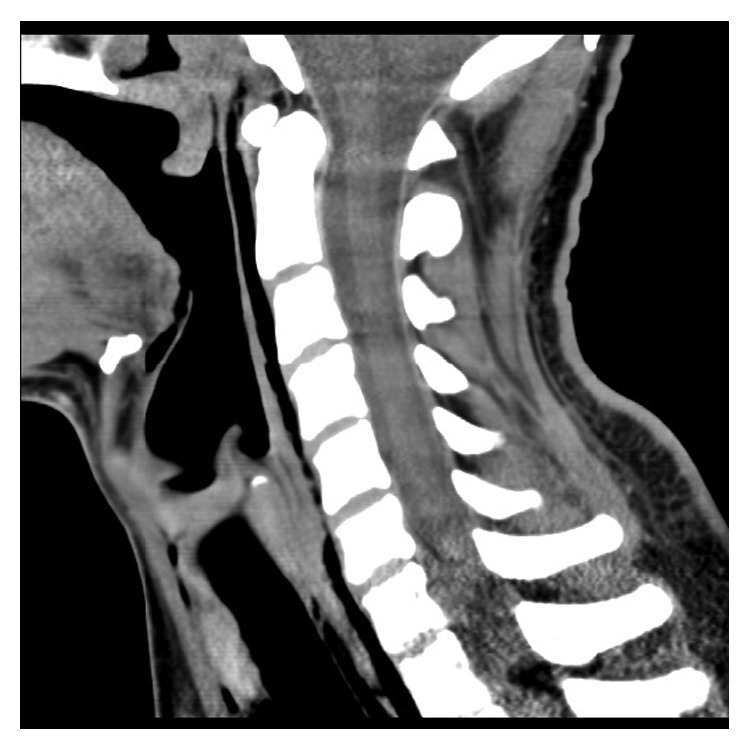
Sagittal CT showing retropharyngeal emphysema extending to the skull base.

On examination, there were no signs of airway compromise, and subcutaneous emphysema was evident in bilateral subclavian regions. Tachycardia, hyperthermia, and hyperreflexia were present. Nasendoscopy showed normal anatomy and airway. Blood tests revealed a mildly raised creatine kinase (CK) of 231 and leukocytes count of 14. Chest X-ray showed significant pneumomediastinum and subcutaneous emphysema (Figure 1) with subsequent computed tomography (CT) showing retropharyngeal emphysema extending from the aortic arch to the base of skull (Figures 2 and 3). There was no evidence of pneumothorax or pneumorrhachis. A gastrografin swallow study showed no contrast extravasation.

He was admitted to hospital for monitoring and conservative management. Follow-up X-ray on day 3 of admission showed resolving pneumomediastinum and subcutaneous emphysema, and he was discharged that same day.

## 3. Discussion

Pneumomediastinum is a rare condition associated with drug abuse. It is well-known to occur secondary to iatrogenic trauma (endoscopy [1] and mechanical ventilation), belching, violent exercise, and underlying pathology (bronchial asthma and emphysema), or it can occur spontaneously [2]. It is termed “spontaneous” when there is no obvious association with intrathoracic visceral perforation [3]. It was first described by Louise Bourgeois in 1617 associated with straining during child birth. When SPM is combined with subcutaneous emphysema, it is referred to as Hamman's Syndrome, a rare condition first described by Louis Virgil Hamman in 1939 [4]. Hamman's sign refers to a precordial crunching sound heard simultaneously with the heart sounds in the presence of pneumomediastinum, caused by the presence of air within the mediastinum as the heart tries to contract [4].

SPM is a very rare sequelae of illicit drug use; however, there are numerous cases in the literature of pneumomediastinum caused by the inhalation of “free-based” or “crack” cocaine (a smoked alkaloid form) [5], where the mechanism of injury is thought to be necrosis and perforation of the posterior pharyngeal wall from the toxic vasoconstrictive properties of the inhaled vapour [5]. There are far fewer cases reported involving nasal insufflation or “snorting” of cocaine powder or the oral ingestion of ecstasy tablets, as in this case. It is also notable that the condition occurred in an otherwise healthy young male and in the absence of heavy physical exertion. Despite our patient administering two different drugs by two different routes, the literature for both drugs suggests the same two mechanisms of injury: marginal alveolar rupture and oesophageal perforation.

**Figure 4:**
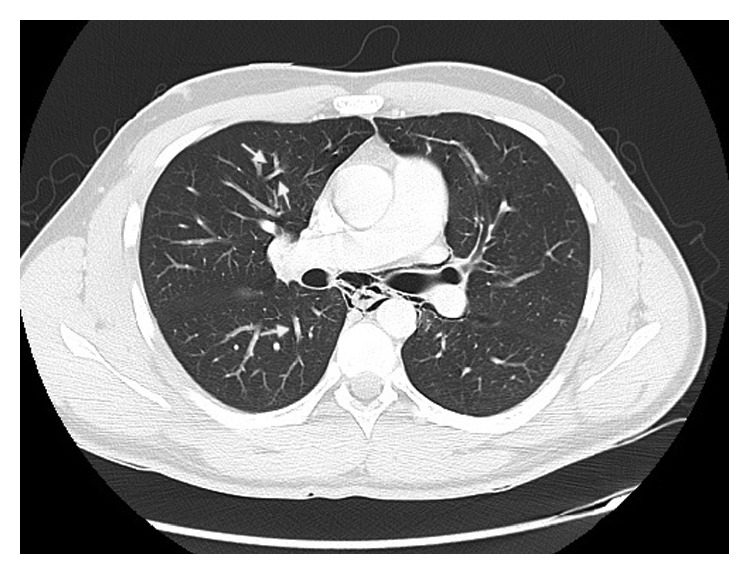
Sagittal CT showing the Macklin effect (arrows) with linear air adjacent to bronchovascular sheaths.

Rupture of marginal pulmonary alveoli can be caused by increased alveolar pressure due to barotrauma. This is known as the Macklin effect and was first described by Macklin and Macklin in 1944 [6]. It is suggested that the decrease in interstitial pressure and increased bronchovascular gradient occurring with forceful inspiration, physical exertion, or prolonged Valsalva manoeuvres can cause a perforation at the marginal bronchial alveolar level or even the tracheobronchial level [7, 8], causing the dissection of free air along vascular planes which provide a path of least resistance. This has been demonstrated on CT as linear collections of air adjacent to bronchovascular sheaths [7], which can be appreciated on our patient's imaging (Figure 4) as well as in a case of pneumomediastinum and pneumorrhachis following ecstasy consumption [9]. The escaped air then reaches the mediastinum resulting in pneumomediastinum [6] before tracking along the deep cervical fascia causing subcutaneous emphysema [10–12], as well as superiorly behind the oesophagus to cause retropharyngeal emphysema. This mechanism is consistent with pneumomediastinum seen in a patient blowing a whistle for 8 hours following ecstasy consumption [10], patients who danced for 12+ hours [8, 13–15], and even a patient who partook in prolonged sexual activity following ingestion of the drug [16]. While alveolar rupture is generally associated with barotrauma, direct toxic effect of the drug causing rupture of the alveoli has also been implicated [11, 17].

The second mechanism is oesophageal perforation. Previous reports have theorised that an oesophageal perforation secondary to barotrauma from vomiting (Boerhaave's syndrome) [3, 18] or even pill dysphagia (and resultant effects of corrosive additives) [2] may explain the presence of mediastinal and retropharyngeal free air. Conditions causing oesophageal dysmotility have been implicated secondarily contributing to increased intraoesophageal pressure [19]. A recent retrospective study identified up to ten percent of patients diagnosed with SPM had a proven oesophageal perforation found on endoscopy, in a contrast study, or discovered during surgery [20]. However, there is only one case in the literature of ecstasy-induced pneumomediastinum which reports a positive gastrografin swallow study [21] and one other case of endoscopy-proven oesophagitis [22]. Ecstasy may also cause gastrointestinal dysmotility which may lead to increased intraluminal pressure and rupture [23].

SPM secondary to drug use usually takes a benign course [10, 12, 24] and should be managed conservatively with 24–48 hours of observation under a surgical (preferably cardiothoracic) team, prophylactic antibiotics, and oxygen therapy [25]. Oxygen acts by washing out nitrogen from mediastinal air pockets and by hastening recovery [11, 26]. Referral to an otolaryngologist for flexible nasendoscopy (FNE) is recommended to look for pharyngeal perforation or injury, followed by a gastrografin swallow in the first 24 hours to assess for oesophageal injury. While extensive workup is not recommended in all cases [25], the combination of the chemical nature and mechanism of administration of the substances involved in drug-related SPM warrants thorough investigation in even the most stable patients [2, 11, 17–19, 23]. The only case in the literature requiring invasive intervention was that of pneumothorax in addition to pneumomediastinum [13], and the only reported case of recurrence of pneumomediastinum was that involving oesophagitis [22].

In a recent Australian survey, 2.2% of responders reported using MDMA in the past 12 months and 2.4% had used cocaine [27].

## 4. Conclusion

Cocaine and ecstasy have both been implicated in the development of SPM and retropharyngeal emphysema by means of direct toxic effects, barotrauma related to methods of ingestion, and euphoria-induced vigorous activity. Given the prevalence of both cocaine and MDMA in our society, this case emphasises the need for an otolaryngologist to take a careful history and examination when assessing patients with cervical subcutaneous emphysema. Appropriate investigations are recommended to exclude serious complications such as oesophageal perforation or pneumothorax.
